# Interplay Among Muscle Oxygen Saturation, Activation, and Power on a Swim-Bench

**DOI:** 10.3390/s25134148

**Published:** 2025-07-03

**Authors:** Vittorio Coloretti, Claudio Quagliarotti, Giorgio Gatta, Maria Francesca Piacentini, Matteo Cortesi, Silvia Fantozzi

**Affiliations:** 1 Department for Life Quality Studies, University of Bologna, 40126 Bologna, Italy; vittorio.coloretti2@unibo.it (V.C.); giorgio.gatta@unibo.it (G.G.); m.cortesi@unibo.it (M.C.); 2 Department of Movement, Human and Health Sciences, University of Rome ‘Foro Italico’, 00135 Rome, Italy; mariafrancesca.piacentini@uniroma4.it; 3 Department of Electrical, Electronic and Information Engineering, University of Bologna, 40126 Bologna, Italy; silvia.fantozzi@unibo.it; 4 Health Science and Technologies Interdepartmental Center for Industrial Research, University of Bologna, 40126 Bologna, Italy

**Keywords:** oximeter, concurrent validity, agreement, sEMG, peripheral fatigue, incremental workload

## Abstract

Muscle activity during exercise is typically assessed using oximeters, to evaluate local oxygen saturation (SmO_2_), or surface electromyography (sEMG), to analyze electrical activation. Despite the importance of combining these analyses, no study has evaluated both of them during specific swimming exercises in combination with mechanical power output. This study aimed to assess muscle activity during an incremental test on a swim-bench utilizing oximeters and sEMG. Nine male swimmers performed a five-steps test: *PRE* (3 min at rest), *STEP* 1, 2, and 3 (swimming at a frequency of 25, 30, and 40 cycle/min for a duration of 2, 2, and 1 min, respectively), and *POST* (5 min at rest). Each swimmer wore two oximeters and sEMG, one for each triceps brachii. Stroke frequency and arm mechanical power (from ~13 to ~52 watts) estimated by the swim-bench were different among all steps, while no differences between arms were found. SmO_2_ (from ~70% to ~60%) and sEMG signals (from ~20 to ~65% in signal amplitude) showed a significant increase among all steps. In both arms, a large/very large correlation was found between mechanical power and SmO_2_ (r < −0.634), mechanical power and sEMG onset/amplitude (r > 0.581), and SmO_2_ and sEMG amplitude (r > 0.508). No correlations were found between the slope of the sEMG spectral indexes and the slope of SmO_2_; only sEMG detected electrical manifestation of muscle fatigue through the steps (*p* < 0.05). Increased muscle activity, assessed by both oximeters and sEMG, was found at mechanical power increases, revealing both devices can detect effort variation during exercise. However, only sEMG seems to detect peripheral manifestations of fatigue in dynamic conditions.

## 1. Introduction

Assessing muscle activity during exercise is crucial for understanding movement patterns, thereby enabling the advancement of biomechanics models, performance optimization, and injury prevention [1,2]. Since muscle activity requires energy production, largely supported by aerobic metabolism in many sports performances, scientists and practitioners in sports science have focused on the assessment of skeletal muscle oxygen delivery and utilization [3,4]. This assessment is normally performed by the evaluation of muscle oxygen saturation (SmO_2_) facilitated by the recent availability of various non-invasive commercial instruments [3,4]. While oximeter sensors have primarily been used to assess SmO_2_ in cycling, running, sport climbing, and resistance training [3], their application in swimming remains limited. Existing swimming studies have focused on evaluating the influence of warm-up or breathing pattern on muscle oxygenation [5,6], as well as studying the relationship between SmO_2_ and other physiological parameters (i.e., heart rate and blood lactate concentration) as an acute effect [7], after different types of recovery [8] or after a training period [9].

As the energy demand for sport performance increases, muscle electrical activation also rises in terms of motor unit recruitment and synchronization [10]. Surface electromyography sensors are used to implement an ecological methodology for measuring muscle electrical activation (sEMG) [2]. In swimming, sEMG was utilized primarily to assess muscle activation patterns in different stroke phases [11], to evaluate electrical manifestation of local muscular fatigue [12], and to analyze muscle synergies [13].

Muscle electrical activation and local muscle oxygenation during swimming may be interconnected, as both reflect the muscle’s energy demand and consumption. As swimming effort increases, the corresponding rise in muscle oxygen consumption, driven by higher energy demands, is linked to greater muscle activity. The distinct physiological insights provided by oximetry and electromyography highlight the need to explore their relationship in greater depth. Integrating SmO_2_ and electrical activation would offer a more holistic and complementary assessment of muscle activity [3]. Indeed, instrumentation including both oximeter and sEMG sensors has been developed [14], and several studies have evaluated muscle oxygenation and activation during strength exercises [14], isometric exercises [15], or dynamic movements (mainly in cycling) [1,14,16,17]. However, no scientific article evaluating muscle activity during simulated swimming exercises by both sensors has been published to date [3], as those undertaken were focused on upper limb and/or on isometric exercises [18]. For water propulsion, it is essential to perform this type of analysis in dynamic controlled conditions, ensuring precise power measurement and movement patterns that closely mimic swimming. In this respect, the swim-bench has been recognized as the most effective tool for dry-land simulated swimming analysis [19].

This study combined the use of oximeters and sEMG for the assessment of the muscle activity of triceps brachii during an incremental test on a swim-bench. The primary objective is to investigate the relationship between muscle oxygen saturation, activation, and power, exploiting SmO_2_, sEMG, and swim-bench, respectively. It was hypothesized that a relationship among these variables will persist even under dynamic conditions and more complex sport-specific movements, such as simulated swimming. Furthermore, since minimal body encumbrance is a required feature for swimming training applications, the measurement agreement between a wearable, wireless oximeter and a wired counterpart was assessed, to control for potential instrumentation-related discrepancies. (see Appendix A).

## 2. Materials and Methods

### 2.1. Participants

The inclusion criteria for the study required participants to be male swimmers specialized in sprint or middle-distance events, with experience in using a swim-bench; while the exclusion criteria were recent muscle or joint injuries, orthopedic, or cardiovascular disease within 3 months before the test.

Nine male swimmers (22.3 ± 2.3 yrs, 181.8 ± 4.9 cm, 76.6 ± 5.2 kg), including five sprinters and four middle-distancers with a personal race best corresponding to 756.4 ± 87.4 World Aquatics points (Level 2–3 [20]) were recruited for the study (see Supplementary Materials for details). All swimmers had been training for at least 12 years, and they had swum, in the last 5 years, a minimum of six days per week, with a training volume ranging between 6 and 7 km per session. They were well-trained in the crawl technique and confident in the use of a swim-bench. Only male and national-level swimmers were included to avoid gender and level biases. Information about the study procedures was provided to each swimmer several days before the data acquisition.

### 2.2. Design

After a standard warm-up of 20 min (simulating dry warm-up before a swimming race and including performing a few minutes at slow pace followed by some spurts on a swim-bench), one incremental step test was performed at the swim-bench by each swimmer wearing two oximeters and two sEMG sensors, one for each triceps brachii. During the test, participants adopted a prone position and were instructed to simulate the front crawl swimming action as closely as possible, including the arm recovery phase. The incremental test was composed of five steps: *PRE* (3 min at rest), *STEP* 1, 2, and 3 (simulated swimming at a frequency of 25, 30, and 40 stroke cycles per minute for durations of 2 min, 2 min, and 1 min, respectively), and *POST* (5 min at rest). A metronome allowed participants to maintain a constant stroke frequency. The swimmers were instructed to be in a fully rested and hydrated state and avoid intensive exercises 48 h before the test.

### 2.3. Methodology

The swimmers performed the test using an integrated swimming machine (swim-bench) for the assessment of arm power output (prototype instrumentation [19,21,22]). The calibration of the air-dynes, both static and dynamic, was carried out following the detailed methodology and features outlined by Zamparo and Swaine [21]. The incremental test was adopted similarly to previous studies conducted with the aim of assessing muscle activation at different intensities [1,23]. The swim-bench measured both stroke frequency and mechanical power expressed by each stroke. The mean value within the central 30 s for each step was used.

Two different near-infrared spectroscopy-derived muscle oxygen saturation sensors were placed in the same position on the right (MOXY, 2 Hz; Fortiori Design LLC, Hutchinson, MN, USA) and left (NIMO, 40 Hz; Nirox srl, Borgosatollo (BS), Italy) arm triceps brachii belly. The sensors were fixed with adhesive and cohesive bands (Fixomull and CoPlus, BSN Medical, Hamburg, Germany) adjacent to the respective sEMG sensor on the same muscle belly, and both were covered with a black plastic bag to prevent exposure to extraneous light. Each oximeter emitting near-infrared light can measure the tissue concentration of oxyhemoglobin (O_2_Hb) and deoxyhemoglobin (HHb). During all the trials, the tissue hemoglobin saturation index (SmO_2_, expressed in % and calculated as [O_2_Hb]/([O_2_Hb] + [HHb]) was directly assessed by the two oximeters.

Two wireless and waterproof surface electromyography (sEMG) sensors (MiniWave, Cometa, Milano, Italy) with a sampling frequency of 2000 Hz were used to assess muscle activation. To enhance the contact between electrodes and the body, the skin of each participant was shaved and abraded following the standards set by SENIAM [24]. Subsequently, Ag/AgCl disposable electrodes (30 × 24 mm) with an active area of 0.8 cm^2^ and an inter-electrode distance of approximately 2 cm were applied in a bipolar configuration. The electrodes were positioned on the belly of the left and right triceps brachii, adjacent to the respective oximeter sensor on the same muscle belly. To optimize signal detection, the surface electrodes were aligned parallel to the direction of the muscle fibers [24]. The sensors were secured to the skin utilizing a cohesive bandage (CoPlus, BSN Medical, Hamburg, Germany) to minimize movement artifacts. The raw signals were: (i) filtered within a band-pass filter (Butterworth, 20–450 Hz), (ii) rectified, and (iii) smoothed using a low-pass filter (6 Hz, 4th order Butterworth) to obtain the linear envelope. The temporal interval of the signal activation interval was assessed for each stroke cycle, and it was expressed as a percentage of the total stroke cycle duration (onset). The mean activation for each stroke cycle onset was expressed as the percentage of the signal maximal value, identified during each step (amplitude). Moreover, to obtain information about the electrical manifestation of local muscular fatigue during the trial, spectral analysis was carried out on each activation interval and the mean and median frequency of the power spectrum of each stroke cycle were identified [25]. Subsequently, the slopes of the mean frequency, median frequency, and SmO_2_ were analyzed. To account for differences in scale between variables, data were normalized: EMG spectral parameters (mean and median frequency) and SmO_2_ were scaled relative to their range values recorded throughout the test. All values are expressed as percentages. The slope of each step was then computed via linear regression to assess the within-step temporal variation of the spectral indices and muscle oxygenation [25].

Thus, at each step, the mean ± standard deviation was estimated in the 30 central seconds (between 45 and 75 in *STEP* 1 and 2, and between 15 and 45 in *STEP* 3) for all the parameters analyzed by the swim-bench, oximeter sensors, and sEMG.

All the analyses discussed were conducted using MatLab (MathWorks, Naticks, MA, USA, 2020b).

### 2.4. Statistical Analysis

The statistical package SPSS version 25.0 (IBM, Chicago, IL, USA) for Windows OS was used for statistical analysis. The significance level was set at *p* ≤ 0.05. Data are presented as the median ± interquartile range.

The non-parametric Friedman test, both with Kendall’s Wallis as the effect size, was performed to assess the differences between steps in the scalar variables, such as stroke frequency, power output, sEMG parameters (amplitude, onset, mean frequency, and median frequency) and SmO_2_. Moreover, the same analysis was performed to compare the slope of the mean/median frequency and the slope of SmO_2_ between the steps and arms. The Wilcoxon test, both with the biserial correlation (r) as effect size, was utilized as a post hoc test in the case of significance in the Friedman test. The non-parametric Mann–Whitney U-test, both with biserial correlation (r) as the effect size, was performed to assess the differences in scalar variables between the right and left arm. The correlation between each measured variable of the same arm was assessed by biserial correlation (r). The value of r was considered as follows: small (0.100–0.299), moderate (0.300–0.499), large (0.500–0.699), very large (0.700–0.899), and extremely large (≥0.900) [26]. Post hoc power analysis was performed utilizing G*Power (version 3.1; Heinrich Heine Universität Düsseldorf). All power analyses had an alpha level of 0.05, nine subjects, and a specific effect size.

## 3. Results

**Figure 1:**
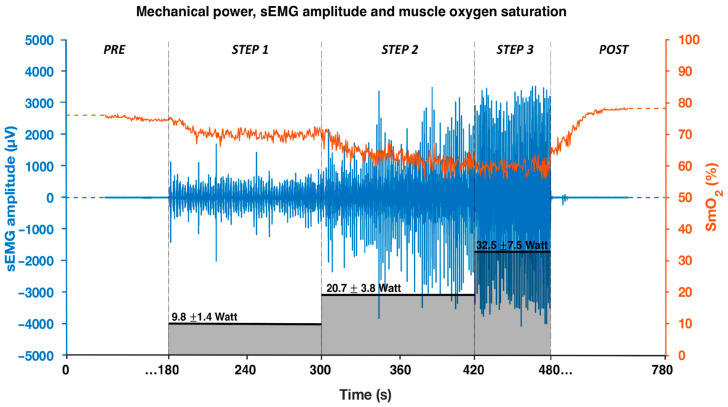
Mechanical power output (black bars), sEMG amplitude signal (blue), and oxygen saturation signal (orange) of the right triceps brachii during the incremental step test. Data refer to a representative swimmer.

Raw data acquired by the sEMG and oximeter sensors during the test are presented together with the mechanical power in Figure 1. As mechanical power increases, a reduction in muscle oxygenation and an increase in muscle activation are observed.

**Figure 2:**
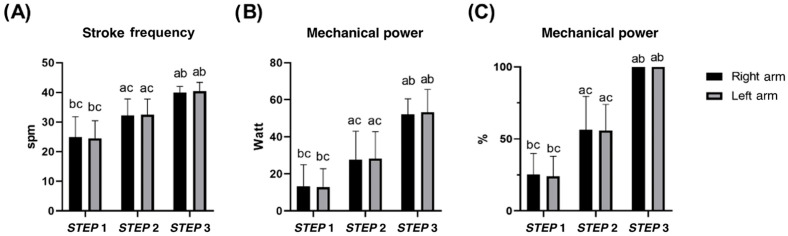
Mean stroke frequency (**A**) and mean mechanical power (**B**,**C**) of each arm in *STEP* 1, 2, and 3 during the incremental step test at the swim-bench. a b c difference between *STEP* 1, 2, and 3, respectively. No differences between left and right arms were found.

Stroke frequency and mechanical power measured by the swim-bench were different among all steps (all *p* < 0.05), and consistent between arms. No differences were observed between arms for both variables (all *p* > 0.05; see Supplementary Materials for details) (Figure 2).

**Figure 3:**
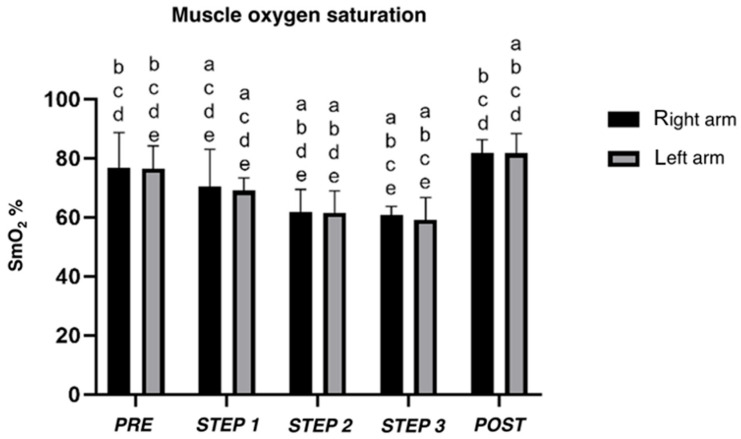
Mean muscle oxygen saturation (SmO_2_) of right and left triceps brachii estimated by oximeters during incremental step test. a, b, c, d, e difference between *PRE*, *STEP* 1, 2, 3, and *POST*, respectively.

Muscle oxygen saturation (Figure 3) decreased between each step for both arms (left arm; *p* < 0.05). Comparing *PRE*- and *POST*-incremental step tests, different results for right (*p* = 0.086) and left arms (*p* = 0.038) were found (see Supplementary Materials for details).

**Figure 4:**
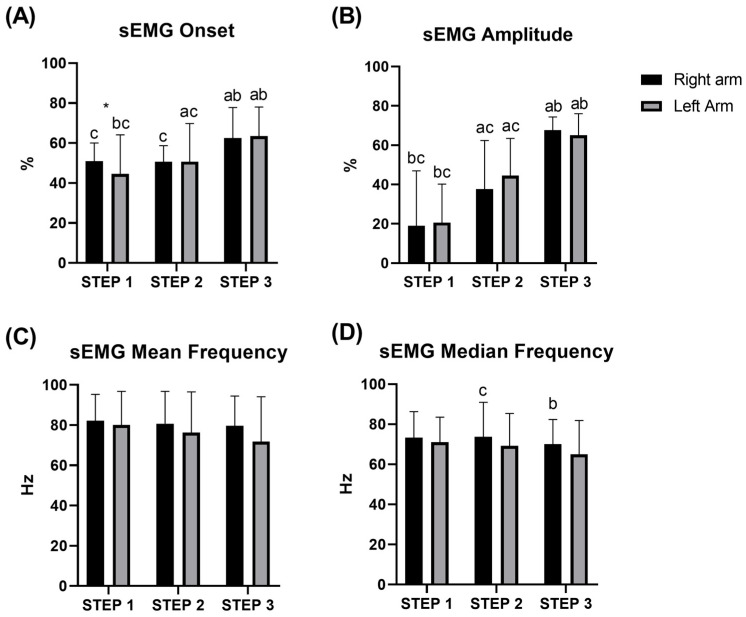
Average of onset (**A**), amplitude (**B**), mean (**C**), and median (**D**) frequency of the sEMG signal of the triceps brachii during *STEP* 1, 2, and 3 during the incremental test at the swim-bench. * difference between left/right arm; a b c significant difference between *STEP* 1, 2, and 3, respectively.

Electromyography signal of the triceps brachii indicated differences between all steps in amplitude and onset (all *p* < 0.05), except for the right arm between *STEP* 1 and 2 (*p* = 0.26). No differences were observed between the left and right arm (all *p* > 0.05), except for *STEP* 1 for the onset (*p* = 0.024) (Figure 4). No differences were detected either between the steps or between the arms, for both the mean frequency and median frequency of the sEMG signal (see Supplementary Materials for details).

**Figure 5:**
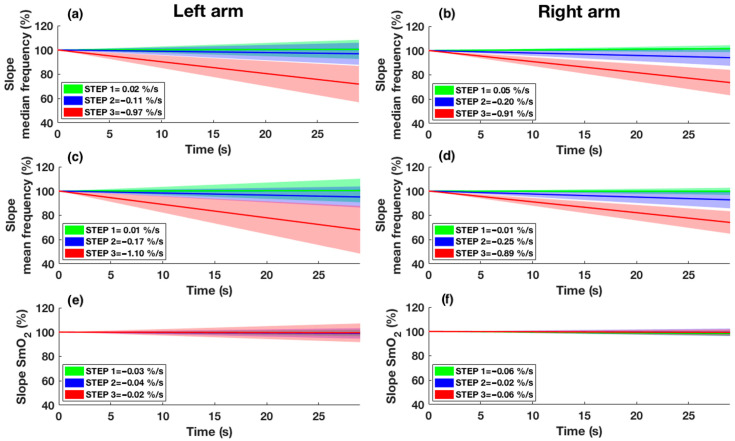
Slope of median frequency (**a**,**b**), slope of mean frequency (**c**,**d**), and slope of SmO_2_ (**e**,**f**) are shown for both arms. *STEP* 1 is represented in green, *STEP* 2 is represented in blue, and *STEP* 3 is represented in red. The continuous line shows the mean slope of the participants in the respective step, while the shade represents the standard deviation. Intercept values with the y-axis of the regression line were set to 100%.

The mean and median frequency slope as well as the SmO_2_ slope are shown in Figure 5. Slope analysis revealed differences between steps for mean (*p* = 0.013 and *p* = 0.001, respectively, for the left and right arm) and median frequency (*p* = 0.004 and *p* = 0.002, respectively, for the left and right arm) and no differences for SmO_2_ (*p* = 0.459 and *p* = 0.895, respectively, for the left and right arm). Post hoc analysis showed differences between *STEP* 1 and 3, and *STEP* 2 and 3 (*p* < 0.05) for the median and mean frequency of both arms, except for the median frequency of the right arm, which showed differences even between *STEP* 1 and 2 (*p* < 0.05). No differences were detected between arms (*p* > 0.05).

Correlations between the mean values of mechanical power, SmO_2_, and sEMG are presented in Table 1 for the right and left arms. Significant correlations were observed between mechanical output (power and stroke frequency) and the sEMG parameters (amplitude and onset), while negative correlations were found between mechanical output (power and stroke frequency) and SmO_2_, or between SmO_2_ and the sEMG amplitude. Correlations between the slope of SmO_2_ and the sEMG spectral indexes are presented in Table 2, where no significant correlations were detected. These correlations were consistent across both arms.

The Appendix A provides information on the concurrent agreement between the wearable, wireless oximeter on the right arm (MOXY), and a cabled gold standard oximeter on the left arm (NIMO), while detailed statistical analysis of power, activation, and muscle oxygenation are available in the Supplementary Materials.

## 4. Discussion

The present study aimed to assess the interplay among muscle oxygenation, activity, and power simultaneously during swimming simulation. As the muscle activation required to generate greater mechanical power increased, a reduction in muscle oxygenation was observed in a controlled incremental exercise on the swim-bench. The relationship between local oxygenation and electrical muscle activity was found only for the amplitude of the EMG signal, but not for the frequency content or the duration of the activation.

The three incremental steps were characterized by different mechanical features. The same mechanical work of both upper limbs was controlled through the use of a metronome and verified by no significant difference between arms in stroke frequency and mechanical power. Under these dynamic controlled conditions of simulated swimming, the results obtained from the two instruments revealed that both oximeters and sEMG are capable of identifying the three distinct exercise intensities. Indeed, a decrease in SmO_2_, meaning an increase in local oxygen consumption, and an increase in both the onset and amplitude of the electromyographic signal are observed for each step. These instruments could, therefore, be useful to obtain more information on exercise intensity, allowing for a more in-depth analysis in the aquatic environment of different swimming techniques [27,28], different recovery profiles and adaptation [9,28,29], or electrical manifestation of muscle fatigue, as well as of the influence of mental fatigue protocols [12,30].

Investigating the relationship among the three different features of muscle, significant correlations were observed between mechanical work and oxygenation, and between mechanical work and all parameters of muscle activation, except for the mean/median frequency. However, the correlation between SmO_2_ and sEMG was significant only for the amplitude feature (r > −0.500, *p* < 0.05). Similarly to the relationship between propulsive phase and swimming velocity in aquatic environment [31], an increase in the percentage duration of the activation phase with higher swimming intensity was observed in this study. However, it seems that among the parameters estimated from EMG, only the signal amplitude is representative of the intensity of the muscle contraction, showing a large correlation with SmO_2_ [32].

Previously, other studies have evaluated the relationship between the information from an oximeter and sEMG during isometric and dynamic exercises. Regarding isometric exercises, similar results to ours were highlighted. Moalla et al. [15] found a negative correlation between muscle oxygenation and the EMG root mean square during isometric knee extension (r = −0.71). Later, Praagman et al. [18] found a linear relationship between oxygen consumption, EMG amplitude, and external loads during isometric contractions of the biceps breve and brachioradialis muscle. In particular, the correlation between oxygen consumption and EMG amplitude was higher than ours for the biceps breve (r = 0.81) and brachioradialis (r = 0.94). Regarding dynamic exercises, in a very similar study to ours, Miura et al. [1] found a high correlation (r = −0.947 to −0.993) in the vastus lateralis muscle between EMG impulse and SmO_2_ during constant work rate (from 50 to 250 watts) on a cycling ergometer. Despite the similarity, the data analyses were different from the present study. For instance, the mean SmO_2_ was compared to the impulse of EMG, both calculated over 30 s of exercise. By contrast, in our work, only the amplitude of the onset part of the signal was considered as the percentage of the maximal force, excluding the signal portion where the muscle was considered deactivated. This procedure allowed us to analyze both the information regarding duration of the activation (i.e., onset) and the intensity of the activation (i.e., amplitude). Moreover, in a more recent study, which utilized EMG and an oximeter during the Astrand–Rhyming Step Test and the Astrand Treadmill Test, a positive correlation was found between data from the oximeter and sEMG with results similar to ours (0.788 and 0.470 for active and non-active participants, respectively) [16]. However, in contrast to the present work, the results derive from the correlation of the HHb and sEMG patterns of the gastrocnemius muscle, instead of the mean SmO_2_ and mean amplitude value of sEMG. In summary, our results appear to agree with previous studies, except for a slightly lower value of the negative correlation. This discrepancy could be attributed to the different approaches in the studies. On one side, because dynamic exercises are more influenced by movement noise than isometric exercises [33], on the other, muscles with a smaller belly may lead to increased difficulty in collecting data [34].

On the other hand, our data showed no differences in the average mean and median frequency of the electromyographic signal throughout the test, thus failing to detect an electrical manifestation of muscle fatigue using this type of approach. Typically, such manifestations are detected through the slope of the spectral indices during isometric contraction [35,36,37], or under constant and high-intensity workloads, as in the case of swimming [12,25]. In the present study, we hypothesized that different workloads might induce different degrees of peripheral muscle fatigue, which would be reflected in the average spectral indices. Accordingly, we applied a simplified method by averaging the spectral EMG indices over the mid-portion of each test step. However, this analytical approach may have lacked sensitivity, masking intra-cycle spectral variability. Indeed, using conventional analysis, a difference in the slope of the regression line between steps was revealed, suggesting that muscle fatigue may manifest differently depending on exercise intensity [38]. Notably, no significant correlation was found between muscle oxygenation assessed via oximeters, and the electrical manifestation of fatigue, either when comparing average values or when evaluating the slopes of the respective regression trends. In fact, although a progressive decline in mean and median EMG frequencies was evident, this was not paralleled by a comparable trend in tissue oxygenation, which exhibited relatively flatter slope profiles.

These results are in contrast with previous studies investigating local fatigue by oximeter and sEMG sensors simultaneously [35,36,37]. However, previously published papers have investigated this relationship during isometric contractions. Taelman et al. [36] observed that, during isometric contractions, a likely factor contributing to muscle fatigue is the constriction of blood vessels and the resulting poor perfusion. This is a physiological condition that does not occur to the same extent during dynamic movements, which may explain the discrepancies observed in comparison with the studies cited. Moreover, it is known how the assessment of dynamic, rather than static, contractions can hide several issues, such as changes in volume conductor properties and electrode position shifts to the analyzed muscle fibers due to variations in joint angle [39]. In contrast, the cyclic nature of the exercise may help in reducing variability and measurement artifacts [39], as reliability in biomechanics is linked to the reliability of the EMG spectral parameters [40].

The present study has some limitations. Considering the limited number of subjects involved, power analysis was performed and showed values higher than 78% (see Supplementary Materials). Another limitation was that only stroke frequency was used to control the intensity of the trial, instead of a percentage of MVC [15,18] or wattage imposed [1]. This was caused by the instrumental limitation of the swim-bench. However, post-control of the stroke frequency and mechanical power showed the incremental work of both arms with no difference in the sides of the body, as well as consistency across subjects. Moreover, the present study analyzed only the triceps brachii muscles, even though other muscles are also particularly involved in the technical swimming act (e.g., latissimus dorsi, pectoralis major, or biceps brachii [2]). Although the triceps brachii is one of the most important and extensively studied muscles in swimming, the lack of analysis of additional muscles may conceal potential compensatory strategies [41]. Consequently, the results and considerations presented in this study should be interpreted taking account of this limitation and cannot be generalized to other muscles. In the present study, only the average value of the middle portion of each step was analyzed to assess the correlation between the sEMG and SmO_2_ parameters due to a lack of direct synchronization of the individual devices used. It would, therefore, be interesting in the future to carry out an instantaneous point-to-point comparison of the various parameters, although in our specific case, all the values were observed to be stable within the middle portion of each step. Finally, the relationship between the EMG amplitude and mechanical power is treated in this work as linear, without considering motor unit derecruitment or selective fatigue of fast-twitch fibers. However, considering that the relationship was based on the mean value of the step, the magnitude of this approximation should be less significant.

In the future, a multi-muscles analysis could help to further understand the degree of utilization and coordination of the different muscles involved during specific movements, as in the case of swimming. In particular, the use of instruments with water-resistant features would allow analysis in the real swimming environment [9,42,43], as in the case of the development of the wireless oximeter used in this study. Moreover, it would also be interesting to investigate in more detail the relationship between the electrical manifestation of muscular fatigue indexes and oxygenation related to peripheral fatigue, which surprisingly showed no interactions. In this context, the individuation of the physiological benchmarks (such as ventilatory/lactate thresholds, %HR or %VO_2_max) may highlight other considerations about the responses of SmO_2_ and sEMG during an effort.

In conclusion, the results obtained indicate that both oximeters and sEMG enable assessment of effort variation during swimming simulation, as already highlighted in isometric and cycling exercises. In particular, it shows that there are correlations between muscle activation and oxygenation during dynamic exercises, or sport-specific exercise as in this case, not only on the lower limbs [16] but also on the upper limbs. Nonetheless, this relationship appears to be confined to the identification of differing effort corresponding to different exercise intensities during the test, whereas only sEMG demonstrated the ability to detect localized fatigue through the electrical manifestations of muscular fatigue. This observation may indicate that, under dynamic conditions, sEMG represents a more suitable tool for the assessment of fatigue manifestation, while the oximeter may be more appropriate for distinguishing between varying levels of exercise intensity.

## Figures and Tables

**Table 1:** Correlation between power output, stroke frequency, muscle oxygen saturation (SmO_2_), and sEMG parameters (onset, amplitude mean, and median frequency) of triceps brachii during incremental step test. * *p* < 0.05. Mechanical power: mechanical power measured by swim-bench; Stroke frequency: stroke frequency measured by swim-bench; SmO_2_: muscle oxygen saturation measured by oximeters; Onset: sEMG onset; Amplitude: sEMG amplitude; Mean frequency: sEMG mean frequency; Median frequency: sEMG median frequency.

	*Right Arm*			*Left Arm*		
	Mechanical Power	Stroke Frequency	SmO_2_	Mechanical Power	Stroke Frequency	SmO_2_
SmO_2_	r = −0.670 *	r = −0.665 *	-	r = −0.634 *	r = −0.612 *	-
Onset	r = 0.581 *	r = 0.564 *	r = −0.283	r = 0.668 *	r = 0.664 *	r = −0.353
Amplitude	r = 0.856 *	r = 0.866 *	r = −0.541 *	r = 0.795 *	r = 0.800 *	r = −0.508 *
Mean frequency	r = −0.353	r = −0.318	r = 0.080	r = −0.212	r = −0.184	r = 0.111
Median frequency	r = −0.350	r = −0.306	r = 0.073	r = −0.216	r = −0.175	r = 0.024

**Table 2:** Correlation between SmO_2_ slope and mean frequency/median frequency slope for each step and both arms. Slope SmO_2_: linear interpolation slope of muscle oxygen saturation measured by oximeters; Slope mean frequency: linear interpolation slope of sEMG mean frequency; Slope median frequency: linear interpolation slope of sEMG median frequency.

	*Right Arm*			*Left Arm*		
	*STEP* 1	*STEP* 2	*STEP* 3	*STEP* 1	*STEP* 2	*STEP* 3
Slope SmO_2_— Slope mean frequency	−0.187	−0.322	−0.595	0.005	−0.384	−0.249
Slope SmO_2_— Slope median frequency	0.001	−0.266	−0.384	0.222	−0.193	−0.434

**Figure A1:**
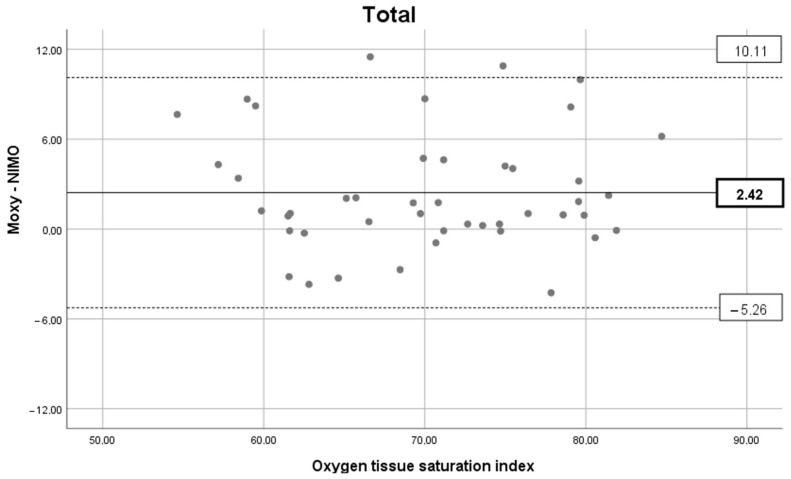
Agreement between MOXY and NIMO data. Bland–Altman plot representing the mean difference (black line) and ± 1.96 × SD (dash lines) considering the total data. Details of agreement between oximeters in each section (*PRE*, *STEP* 1, *STEP* 2, *STEP* 3, and *POST*) are available in the Supplementary Materials.

## Data Availability

The data presented in this study are available upon request from the corresponding authors. The data are not publicly available due to privacy restrictions.

## Supplementary Materials

The following supporting information can be downloaded at: https://www.mdpi.com/article/10.3390/s25134148/s1.

## Appendix A. Concurrent Agreement Between NIMO and MOXY Oximeter Sensors

### Appendix A.1. Introduction

In the past two decades, NIRS technology has aroused increasing interest in the field of sports science thanks to its use in the non-invasive monitoring of hemodynamics and muscle oxygenation [3,4]. However, several limitations associated with NIRS-based oximetry have been extensively highlighted in recent literature reviews [34,44,45]. These include, for instance, the influence of subcutaneous adipose tissue overlying the muscle [46], variations in melanin content [47], differential skin perfusion in response to exercise [48], and sensor displacement or artifacts caused by muscle shortening during contractions [4]. Additionally, the relatively small sampling volume and shallow penetration depth of NIRS may further compromise measurement accuracy and subsequent interpretation of the total work of the muscle [34].

These complexities are exacerbated by the existence of various NIRS devices, which differ in multiple aspects, such as size, number, and spacing of emitters and detectors, and operational wavelengths [34,44,45]. Moreover, NIRS systems rely on different technological principles, including Continuous Wave (CW), Frequency Domain (FD), and Time Domain (TD) methods [34,45,49,50]. While CW-NIRS devices are less expensive and more user-friendly, as well as having mostly wireless features that lend themselves to easy application in the sports field, they present some notable limitations compared to FD and TD systems. FD and TD instruments offer superior depth discrimination and allow for the absolute quantification of tissue optical properties, such as absorption and reduced scattering coefficients. From these, absolute concentrations of [oxy(Hb + Mb)] and [deoxy(Hb + Mb)] can be derived, along with the total concentration [total(Hb + Mb)]. In contrast, currently available CW-NIRS instruments do not measure the reduced scattering coefficient directly, and instead assume it to be relatively constant during both rest and exercise conditions [34,45,49,50]. As a result, CW devices are unable to provide true absolute oxygenation values and are more susceptible to the previously mentioned sources of artifact [28,34,49,51]. Despite the cited limitations, NIRS technology, and in particular, CW-NIRS, deserves further investigation as it still represents a promising solution for monitoring oxygenation in clinical and sports contexts [28].

Several studies have compared the performance of different NIRS devices and examined their measurement reliability and agreement, though findings remain inconsistent [23,51,52,53,54,55]. Notably, the MOXY device has been more recently developed, including a new version for aquatic performance monitoring. Although MOXY has shown high reliability and validity [56] and moderate inverse correlation with metabolic oxygen consumption VO_2_ [57,58], when compared to other CW-NIRS devices (i.e., Portamon, Humon Hex and NIMO), it has shown both favorable [52] and unfavorable [23,51] outcomes. In more detail, comparisons between MOXY and the NIMO device have been conducted under static conditions involving only vascular occlusion, detecting changes in the SmO_2_ percentage but without assessing performance in dynamic exercise conditions [51]. In general, there is an accordance when vascular occlusion calibration is performed to compensate for differences between devices, and a weaker accordance with motor exercise at low intensities. Considering the literature findings, the agreement analysis must be performed in the specific context of swimming simulation.

Therefore, the aim of this parallel study is to compare the performance of the MOXY and NIMO devices under dynamic conditions, in order to assess the agreement between their respective measurements of muscle oxygenation.

### Appendix A.2. Method

The two oximeters utilized in this study were MOXY (Hutchinson, MN, USA) and NIMO (Nirox, Borgosatollo (BS), Italy). The characteristics of the devices are reported in a previous comparison study, which analyzed their performance during an arterial occlusion [59].

Data were acquired during the protocol explained in the main text. NIMO raw data were resampled from 40 Hz to 2 Hz. Moreover, a physiological calibration after arterial occlusion was performed by applying a hemostatic elastic to the proximal part of the humerus for 5 min just after the test, followed by 5 min of rest without any occlusion [23]. The minimum and maximum SmO_2_ values were measured averaging 30 s of data at the end of deoxygenation and after reoxygenation, when the SmO_2_ value stabilized. These values obtained from the occlusion task were subsequently used to establish notional 0%, 100% and Δ% of each instrument [23]. Subsequently, the SmO_2_ values recorded during the incremental test were rescaled according to the new range derived from the occlusion task in order to compare the two devices’ output.

The rescaled data from the two oximeters were then compared at each different step intensity in terms of concurrent validity and agreement. Thus, biserial correlation (r) was performed and Bland–Altman plots were constructed, respectively. The differences between devices were calculated as the mean of the oxygen tissue saturation index of MOXY minus NIMO. The value of r was considered as follows: small (0.100–0.299), moderate (0.300–0.499), large (0.500–0.699), very large (0.700–0.899), and extremely large (≥0.900) [26].

### Appendix A.3. Results

The concurrent validity between MOXY and NIMO sensors was significant in Total (r = 0.911, *p* = 0.000) at *PRE* (r = 0.804, *p* = 0.009), *STEP* 3 (r = 0.859, *p* = 0.003), *POST* (r = 0.792, *p* = 0.011), and all Steps (r = 0.806, *p* = 0.000), but not at *STEP* 1 (r = 0.501, *p* = 0.170) and *STEP* 2 (r = 0.643, *p* = 0.062). The agreement between the MOXY and NIMO data in Total, at *PRE*, *STEP* 1, 2, 3, and *POST* is presented in Figure A1. No differences in SmO_2_ (Figure 2) between MOXY (right arm) and NIMO (left arm) were found within each step (all *p* > 0.05).

### Appendix A.4. Discussion

The secondary aim of the project was to test the concurrent validity and agreement between two different NIRS. The results show that concurrent validity was significant with a very large/extremely large correlation coefficient in *PRE*, *STEP* 3, *POST*, all-Steps, and Total (≥0.804). On the other hand, no significant correlation was found for *STEP* 1 and *STEP* 2. In addition, the Bland–Altman analysis reflects a lower agreement between the two oximeters during the first two steps of intensity, with large limits of agreement found. These results are consistent with a previous comparison study concerning MOXY and Portamon, which evidenced low concurrent validity and agreement during a low/moderate intensity incremental arm-cranking task [23]. However, as already explained [3], the discrepancy in the measurements obtained from different NIRS instruments can be attributed primarily to the variations in the underlying technologies and algorithms employed. This elucidates the reason for the disparate absolute O_2_Hb and HHb concentration values or SmO_2_ ranges. Nevertheless, by performing an arterial occlusion and rescaling the values obtained through a process of ‘physiological calibration’, it is possible to achieve more comparable measurements [23]. Notwithstanding this, the uncertainty in the measurement persists, which may be attributed also to the disparate positions of the sensor or body area measured, the thickness of the surface fat, or the pressure exerted to secure the device [34]. All these factors must be taken into account when working with NIRS devices.

On the other hand, a recent study has found an excellent correlation and no significant differences in the evaluation of vastus lateralis oxygenation during a running test at sub-maximal intensity [52]. The study, where MOXY and Humon Hex were employed, showed no significant differences in measuring SmO_2_ and a significant correlation for each step (*p* > 0.577). However, even in this case, the authors pointed out that there were differences in oxygenation of up to 5%, and that therefore, although the instruments showed agreement, caution should be taken when measuring oxygenation with different devices [52]. In another comparison study between MOXY and NIMO [59], different absolute SmO_2_ values were found during an arterial occlusion. Therefore, the authors highlighted how the two devices lead to differences in oxygenation and perfusion measurements. It is noteworthy that a higher measurement range in the MOXY than in other devices has also been shown in other studies [23], where changes in oxygenation of 74.3% for the MOXY and 43.7% for the Portamon were found following occlusion. However, it is by normalizing to this range of variability that the measurements made by the devices become comparable during dynamic exercise, as anticipated above [23].

Therefore, in light of the mechanical power and stroke frequency values expressed by the arms during the tests, as well as the notable difference in intensity between one step and the next, it can be stated that the two devices analyzed in this work (MOXY and NIMO) experienced a change in oxygenation at each step, demonstrating their ability to perceive the varying levels of exertion during an incremental test. It should be noted, however, that the measurements taken are not directly comparable, and that caution should be exercised when interpreting the results from different NIRS devices [60]. Finally, it is worth noting that the main objective of this paper was to compare the interaction between muscle oxygen saturation, activity, and power, comparing the values just in the unilateral condition. As a consequence, the discrepancy between the two devices highlighted here does not affect the main findings presented.
