# Supplementary material for: Interplay Among Muscle Oxygen Saturation, Activation, and Power on a Swim-Bench
Source: Sensors (Basel). 2025 Jul 3;25(13):4148. doi: 10.3390/s25134148 (PMC12252474; doi:10.3390/s25134148)
Supplement: Supplementary file 1 [file sensors-25-04148-s001.zip › sensors-3590268-supplementary.pdf]

Supplementry Materials

Detailed data

Subjects details

Subject	Discipline	World Acquatics point
1	200 butterfly	791
2	200 butterfly	774
3	400 medley	814
4	200 front crawl	861
5	100 front crawl	840
6	100 breaststroke	712
7	50 butterfly	562
8	100 front crawl	774
9	200 backstroke	710

In the following tables are presented detailed data analysed (Median±Interquartile range)

Stroke frequency

	Step-1	Step-2	Step-3
**Right arm**	24.93±6.87[b,c]	32.19±5.57[a,c]	39.93±2.08[a,b]
**Left Arm**	24.49±5.93 [b,c]	32.50±5.23 [a,c]	40.39±3.02 [a,b]

[a b c] significant different between STEP 1, 2 and 3, respectively. No differences between left and right arm were found.

Mechanical power

	Step-1	Step-2	Step-3
**Right arm**	13.38±11.60 W [b,c]	27.61±15.42 W [a,c]	51.11±8.41 W [a,b]
**Left Arm**	12.81±9.94 W [b,c]	28.33±14.47 W [a,c]	53.27±12.35 W [a,b]

[a b c] significant different between STEP 1, 2 and 3, respectively. No differences between left and right arm were found.

Mechanical power

	Step-1	Step-2	Step-3
**Right arm**	25.26±14.54 % [b,c]	56.39±23.07 % [a,c]	100 % [a,b]
**Left Arm**	24.05±13.86 % [b,c]	55.76±18.20 % [a,c]	100 % [a,b]

[a b c] significant different between STEP 1, 2 and 3, respectively. No differences between left and right arm were found.

sEMG Onset

	Step-1	Step-2	Step-3
**Right arm**	50.99±9.03 %[c, *]	50.67±8.00 %[c]	62.56±15.26 %[a,b]
**Left Arm**	44.51±19.58 %[b,c,*]	50.74±19.08 %[a,c]	63.44±14.93 %[a,b]

[a b c] significant difference between STEP 1, 2 and 3, respectively. * significant difference between arms

sEMG Amplitude

	Step-1	Step-2	Step-3
**Right arm**	18.97±27.95 %[b,c]	37.60±24.79 %[a,c]	67.61±6.80 %[a,b]
**Left Arm**	20.61±19.58 %[b,c]	44.52±18.96 %[a,c]	65.04±10.97 %[a,b]

[a b c] significant different between STEP 1, 2 and 3, respectively. No differences between left and right arm were found.

sEMG mean frequency

	Step-1	Step-2	Step-3
**Right arm**	82.20±13,10 Hz	80.70±16.10 Hz	79.7±14.75 Hz

**Left Arm**	80.10±16.65 Hz	76.30±20.25 Hz	71.80±22.35 HZ

No differences between left and right arm or between Step were found.

sEMG median frequency

	**Step-1**	**Step-2**	**Step-3**
**Right arm**	73.30±12.95 Hz	73.70±17.25 Hz	70.00±12.35 Hz
**Left Arm**	71.00±12.50 Hz	69.20±16.20 Hz	65.00±16.90 Hz

No differences between left and right arm or between Step were found.

Muscle Oxygenation Saturation

	**Pre**	**Step-1**	**Step-2**	**Step-3**	**Post**
**Right arm**	76.77±11.90 %	70.34±8.36 %	61.77±7.70 %	60.72±2.96 %	81.88±4.38 %
**Left Arm**	76.52±7.65 %	69.16±4.24 %	61.54±7.42 %	59.16±7.51 %	81.78±6.58 %

[a b c d e] significant different between PRE, STEP 1, 2, 3 and POST respectively. No differences between left and right arm were found.

Slope of sEMG median frequency

	**Step-1**	**Step-2**	**Step-3**
**Right arm**	.09±.18	-.22±.39	-1.07±.67
**Left Arm**	.02±.33	.00±.59	-1.02±.87

No differences between left and right arm or between Step were found.

Slope of sEMG mean frequency

	**Step-1**	**Step-2**	**Step-3**
**Right arm**	.00±.16	-.29±.39	-.96±.55
**Left Arm**	.02±.54	-.01±.58	-1.10±1.29

No differences between left and right arm or between Step were found.

Slope of Muscle Oxygenation Saturation

	**Step-1**	**Step-2**	**Step-3**
**Right arm**	-.06±.08	.01±.19	-.03±.13
**Left Arm**	-.04±.13	-.01±.24	-0.06±.25

No differences between left and right arm or between Step were found.

Statistical Analysis

In the following tables are presented detailed statistics

Within arms

Frequency – Right

$X^2_{(9,2)}$=18.000 p=.000* W=1.000 Power=.999

	Step 1	Step 2	Step 3
Step 1		Z=-2.666 p=.008* r=.714 Power=.448	Z=-2.666 p=.008* r=.664 Power=.398
Step 2	Z=-2.666 p=.008* r=.714 Power=.448		Z=-2.666 p=.008* r=.799 Power=.533
Step 3	Z=-2.666 p=.008* r=.664 Power=.398	Z=-2.666 p=.008* r=.799 Power=.533	

Frequency – Left

$X^2_{(9,2)}$=18.000 p=.000* W=1.000 Power=.999

	Step 1	Step 2	Step 3
Step 1		Z=-2.666 p=.008* r=.771 Power=.505	Z=-2.666 p=.008* r=.803 Power=.537
Step 2	Z=-2.666 p=.008* r=.771 Power=.505		Z=-2.666 p=.008* r=.633 Power=.368
Step 3	Z=-2.666 p=.008* r=.803 Power=.537	Z=-2.666 p=.008* r=.633 Power=.368	

Power output – Right

$X^2_{(9,2)}$=18.000 p=.000* W=1.000 Power=.999

	Step 1	Step 2	Step 3
Step 1		Z=-2.666 p=.008* r=.679 Power=.413	Z=-2.666 p=.008* r=.610 Power=.347
Step 2	Z=-2.666 p=.008* r=.679 Power=.413		Z=-2.666 p=.008* r=.820 Power=.554
Step 3	Z=-2.666 p=.008* r=.610 Power=.347	Z=-2.666 p=.008* r=.820 Power=.554	

Power output – Left

$X^2_{(9,2)}$=18.000 p=.000* W=1.000 Power=.999

	Step 1	Step 2	Step 3

Step 1		Z=-2.666 p=.008* r=.807 Power=.541	Z=-2.666 p=.008* r=.800 Power=.534
Step 2	Z=-2.666 p=.008* r=.807 Power=.541		Z=-2.666 p=.008* r=.651 Power=.386
Step 3	Z=-2.666 p=.008* r=.800 Power=.534	Z=-2.666 p=.008* r=.651 Power=.386	

Power output percentage – Right

$X^2_{(9,2)}$=18.000 p=.000* W=1.000 Power=.999

	Step 1	Step 2	Step 3
Step 1		Z=-2.666 p=.008* r=.506 Power=.255	Z=-2.666 p=.008* r=.
Step 2	Z=-2.666 p=.008* r=.506 Power=.255		Z=-2.666 p=.008* r=.
Step 3	Z=-2.666 p=.008* r=.	Z=-2.666 p=.008* r=.	

Power output percentage – Left

$X^2_{(9,2)}$=18.000 p=.000* W=1.000 Power=.999

	Step 1	Step 2	Step 3
Step 1		Z=-2.666 p=.008* r=.629 Power=.364	Z=-2.666 p=.008* r=.
Step 2	Z=-2.666 p=.008* r=.807 Power=..541		Z=-2.666 p=.008* r=.
Step 3	Z=-2.666 p=.008* r=.	Z=-2.666 p=.008* r=.	

Onset– Right

$X^2_{(9,2)}$=8.222 p=.016* W=.457 Power=.785

	Step 1	Step 2	Step 3
Step 1		Z=-1.125 p=.260 r=.195 Power=.080	Z=-2.192 p=.028* r=-.279 Power=.111
Step 2	Z=-1.125 p=.260 r=.195 Power=.080		Z=-2.666 p=.008* r=.717 Power=.451
Step 3	Z=-2.192 p=.028* r=-.279 Power=.111	Z=-2.666 p=.008* r=.717 Power=.451	

Onset – Left

$X^2_{(9,2)}$=16.222 p=.000* W=.901Power=.999

	Step 1	Step 2	Step 3
Step 1		Z=-2.249 p=.015* r=.819 Power=.553	Z=-2.666 p=.008* r=.626 Power=.362
Step 2	Z=-2.429 p=.015* r=.819 Power=.553		Z=-2.666 p=.008* r=.885 Power=.618
Step 3	Z=-2.666 p=.008* r=.626 Power=.362	Z=-2.666 p=.008* r=.885 Power=.618	

Amplitude– Right

$X^2_{(9,2)}$=18.000 p=.000* W=1.000 Power=.999

	Step 1	Step 2	Step 3
Step 1		Z=-2.666 p=.008* r=.906 Power=.638	Z=-2.666 p=.008* r=.043 Power=.051
Step 2	Z=-2.666 p=.008* r=.906 Power=.638		Z=-2.666 p=.008* r=.096 Power=.057
Step 3	Z=-2.666 p=.008* r=.043 Power=.051	Z=-2.666 p=.008* r=.096 Power=.057	

Amplitude – Left

$X^2_{(9,2)}$=18.000 p=.000* W=1.000 Power=.999

	Step 1	Step 2	Step 3
Step 1		Z=-2.666 p=.008* r=.879 Power=.613	Z=-2.666 p=.008* r=.194 Power=.079
Step 2	Z=-2.666 p=.008* r=.879 Power=.613		Z=-2.666 p=.008* r=.457 Power=.217
Step 3	Z=-2.666 p=.008* r=.194 Power=.079	Z=-2.666 p=.008* r=.457 Power=.217	

Mean frequency – Right

$X^2_{(9,2)}$=4.222 p=.121 W=.235 Power=.265

Mean frequency – Left

$X^2_{(9,2)}$=.889 p=.641 W=.049 Power=.058

Median frequency – Right

$X^2_{(9,2)}$=8.667 p=.013* W=.481 Power=.816

	Step 1	Step 2	Step 3
Step 1		Z=-1.362 p=.173 r=.933 Power=.663	Z=-1.838 p=.066 r=.425 Power=.194
Step 2	Z=-1.362 p=.173 r=.933 Power=.663		Z=-2.194 p=.028* r=.573 Power=.313
Step 3	Z=-1.838 p=.066 r=.425 Power=.194	Z=-2.194 p=.028* r=.573 Power=.313	

Median frequency – Left

$X^2_{(9,2)}$=2.667 p=.264 W=.148 Power=.129

Slope Mean frequency – Right

$X^2_{(9,2)}$=14.000 p=.001* W=.778 Power=.998

	Step 1	Step 2	Step 3
Step 1		Z=-1.955 p=.051 r=-.432 Power=.199	Z=-2.668 p=.008* r=-.360 Power=.153
Step 2	Z=-1.955 p=.051 r=-.432 Power=.199		Z=-2.666 p=.008* r=.231 Power=.092
Step 3	Z=-2.668 p=.008* r=-.360 Power=.153	Z=-2.666 p=.008* r=.231 Power=.092	

Slope Mean frequency – Left

$X^2_{(9,2)}$=8.667 p=.013* W=.489 Power=.840

	Step 1	Step 2	Step 3
Step 1		Z=-1.125 p=.260 r=-.266 Power=.105	Z=-2.429 p=.015* r=-627. Power=.363
Step 2	Z=-1.125 p=.260 r=-.266 Power=.105		Z=-2.547 p=.011* r=-.154 Power=.068
Step 3	Z=-2.429 p=.015* r=-627. Power=.363	Z=-2.547 p=.011* r=-.154 Power=.068	

Slope Median frequency – Right

$X^2_{(9,2)}$=12.667 p=.002* W=.704 Power=.991

	Step 1	Step 2	Step 3
Step 1		Z=-2.192 p=.028* r=-.372 Power=.160	Z=-2.666 p=.008* r=.161 Power=.070
Step 2	Z=-2.192 p=.028* r=-.372 Power=.160		Z=-2.549 p=.011* r=.046 Power=.052
Step 3	Z=-2.666 p=.008* r=.161 Power=.070	Z=-2.549 p=.011* r=.046 Power=.052	

Slope Median frequency – Left

$X^2_{(9,2)}$=10.889 p=.004* W=.605 Power=.959

	Step 1	Step 2	Step 3
Step 1		Z=-.533 p=.594 r=-.460 Power=.219	Z=-2.666 p=.008* r=-.376 Power=.162
Step 2	Z=-.533 p=.594 r=-.460 Power=.219		Z=-2.547 p=.011* r=-.266 Power=.105
Step 3	Z=-2.666 p=.008* r=-.376 Power=.162	Z=-2.547 p=.011* r=-.266 Power=.105	

Slope SmO2 – Right

$X^2_{(9,2)}$=.222 p=.895 W=.012 Power=.050

Slope SmO2 – Left

$X^2_{(9,2)}$=1.556 p=.459 W=.086 Power=.076

SmO_2 – Moxy (right)

$X^2_{(9,4)}$=34.044 p=.000* W=.946 Power=.999

	Pre	Step 1	Step 2	Step 3	Post
Pre		Z=-2.666 p=.008* r=.653 Power=.370	Z=-2.666 p=.008* r=.425 Power=.194	Z=-2.666 p=.008* r=.064 Power=.053	Z=-1.718 p=.086 r=.722 Power=.456
Step 1	Z=-2.666 p=.008* r=.653 Power=.370		Z=-2.666 p=.008* r=.917 Power=.648	Z=-2.666 p=.008* r=.597 Power=.334	Z=-2.666 p=.008* r=.270 Power=.107
Step 2	Z=-2.666 p=.008* r=.425 Power=.194	Z=-2.666 p=.008* r=.917 Power=.648		Z=-2.192 p=.028* r=.811 Power=.545	Z=-2.666 p=.008* r=.047 Power=.052
Step 3	Z=-2.666 p=.008* r=.064 Power=.053	Z=-2.666 p=.008* r=.597 Power=.334	Z=-2.192 p=.028* r=.811 Power=.545		Z=-2.666 p=.008* r=-.085 Power=.056
Post	Z=-1.718 p=.086 r=.722 Power=.456	Z=-2.666 p=.008* r=.270 Power=.107	Z=-2.666 p=.008* r=.047 Power=.052	Z=-2.666 p=.008* r=-.085 Power=.056	

SmO_2 – NIMO (left)

$X^2_{(9,4)}$=33.778 p=.000* W=.938 Power=.999

	Pre	Step 1	Step 2	Step 3	Post
Pre		Z=-2.310 p=.021* r=.260 Power=.102	Z=-2.666 p=.008* r=.096 Power=.058	Z=-2.666 p=.008* r=-.031 Power=.051	Z=-2.073 p=.038* r=.585 Power=.323
Step 1	Z=-2.310 p=.021* r=.260 Power=.102		Z=-2.666 p=.008* r=.939 Power=.669	Z=-2.666 p=.008* r=.866 Power=.600	Z=-2.666 p=.008* r=-.016 Power=.050
Step 2	Z=-2.666 p=.008* r=.096 Power=.058	Z=-2.666 p=.008* r=.939 Power=.669		Z=-2.547 p=.011* r=.924 Power=.655	Z=-2.666 p=.008* r=-.220 Power=.088

Step 3	Z=-2.666 p=.008* r=-.031 Power=.051	Z=-2.666 p=.008* r=.866 Power=.600	Z=-2.547 p=.011* r=.924 Power=.655		Z=-2.666 p=.008* r=-.082 Power=.055
Post	Z=-2.073 p=.038* r=.585 Power=.323	Z=-2.666 p=.008* r=-.016 Power=.050	Z=-2.666 p=.008* r=-.220 Power=.088	Z=-2.666 p=.008* r=-.082 Power=.055	

Between arms

	Step 1	Step 2	Step 3
Frequency	$U_{(18)}$=37.000 p=.757 r=-.033 Power=.050	$U_{(18)}$=38.000 p=.825 r=.048 Power=.051	$U_{(18)}$=29.000 p=.310 r=.165 Power=.062
Power output	$U_{(18)}$=37.000 p=.757 r=-.057 Power=.051	$U_{(18)}$=37.000 p=.757 r=.033 Power=.050	$U_{(18)}$=30.000 p=.35 r=.121 Power=.056
Power output %	$U_{(18)}$=35.000 p=.627 r=-.129 Power=.057	$U_{(18)}$=39.000 p=.895 r=-.017 Power=.050	$U_{(18)}$=40.500 p=1.000 r=1.000 Power=.722
Onset	$U_{(18)}$=15.000 p=.024* r=-.515 Power=.170	$U_{(18)}$=34.000 p=.566 r=-260 Power=.080	$U_{(18)}$=32.000 p=.453 r=-.205 Power=.068
Amplitude	$U_{(18)}$=40.000 p=.965 r=-.056 Power=.051	$U_{(18)}$=38.000 p=.825 r=.069 Power=.052	$U_{(18)}$=26.000 p=.200 r=-.364 Power=.109
Mean Frequency	$U_{(18)}$=38.000 p=.825 r=-.183 Power=.065	$U_{(18)}$=29.000 p=.310 r=-.251 Power=.078	$U_{(18)}$=29.000 p=.310 r=-.263Power=.080
Median Frequency	$U_{(18)}$=34.000 p=.566 r=-.117 Power=.056	$U_{(18)}$=29.000 p=.310 r=-.290 Power=.087	$U_{(18)}$=31.000 p=.402 r=-.263 Power=.080
Slope Mean Frequency	$U_{(18)}$=39.000 p=.895 r=.711 Power=.282	$U_{(18)}$=31.500 p=.426 r=.482 Power=.155	$U_{(18)}$=37.000 p=.757 r=.124 Power=.057
Slope Median Frequenc	$U_{(18)}$=33.500 p=.536 r=.453 Power=.142	$U_{(18)}$=34.000 p=.566 r=.389 Power=.118	$U_{(18)}$=40.000 p=.965 r=.372 Power=.112
Slope SmO2	$U_{(18)}$=31.500 p=.425 r=-.216 Power=.070	$U_{(18)}$=36.000 p=.691 r=.629 Power=.231	$U_{(18)}$=27.000 p=.232 r=.542 Power=.184

	Pre	Step 1	Step 2	Step 3	Post
SmO_2	$U_{18)}$=30.000 p=.354 r=-.293 Power=.088	$U_{18)}$=31.000 p=.402 r=-.264 Power=.081	$U_{18)}$=32.000 p=.453 r=-.170 Power=.063	$U_{18)}$=28.000 p=.270 r=-.212 Power=.070	$U_{18)}$=38.000 p=.825 r=-.150 Power=.060

Bland-Altman plots

In the following figures are presented agreement analyses between Moxy and Nimo at each step.

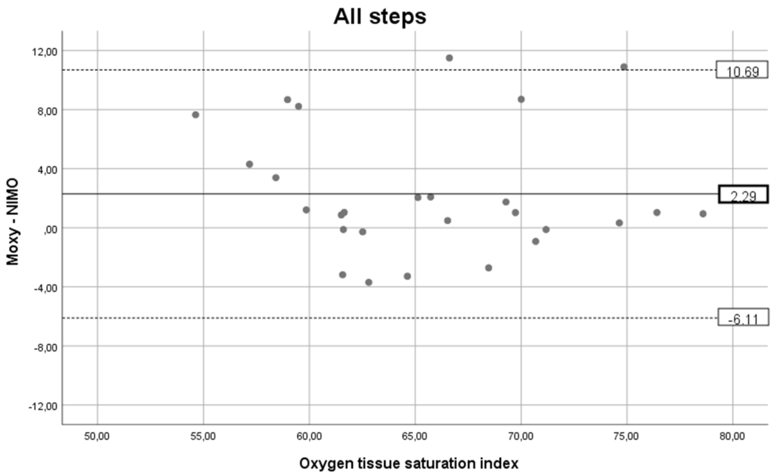

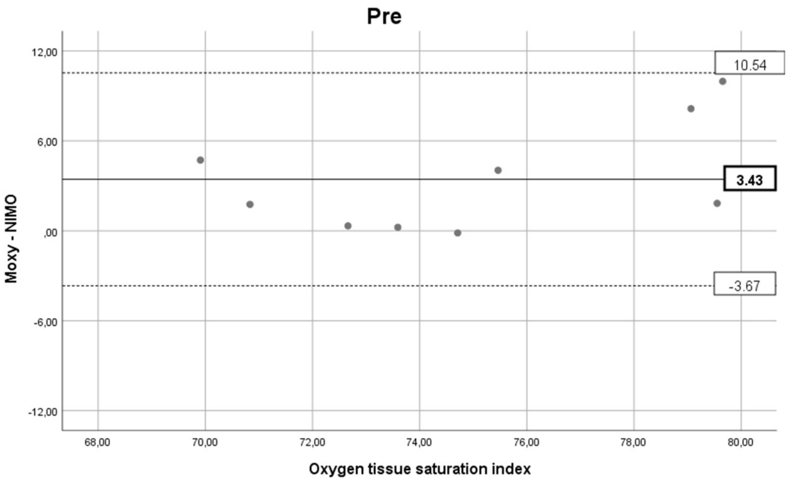

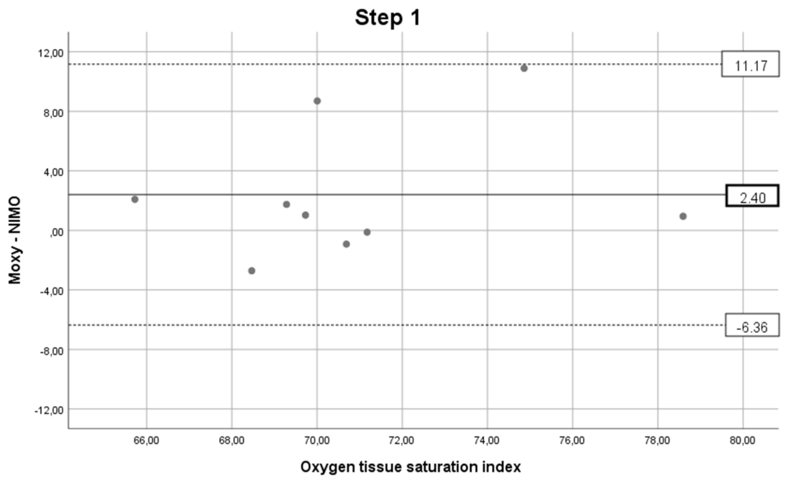
Step 1
12,00
8,00
4,00
,00
-4,00
-8,00
-12,00
11.17
2.40
-6.36
Moxy - NIMO
66,00
68,00
70,00
72,00
74,00
76,00
78,00
80,00
Oxygen tissue saturation index

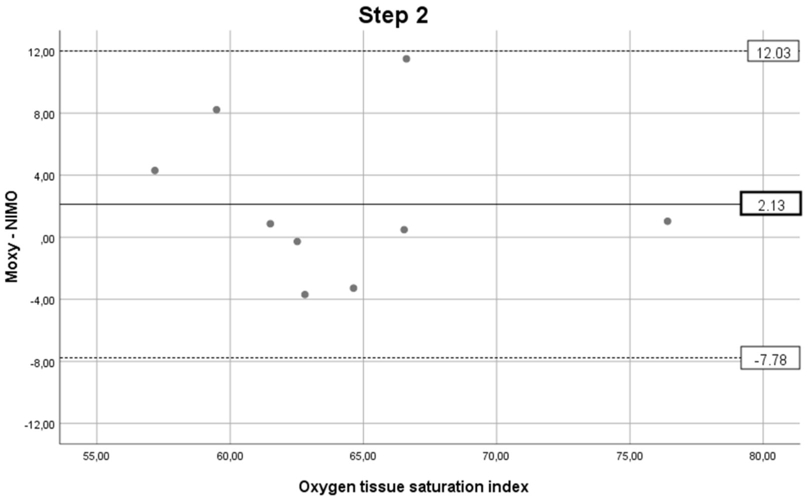
Step 2
12,00
8,00
4,00
,00
-4,00
-8,00
-12,00
12.03
2.13
-7.78
Moxy - NIMO
55,00
60,00
65,00
70,00
75,00
80,00
Oxygen tissue saturation index

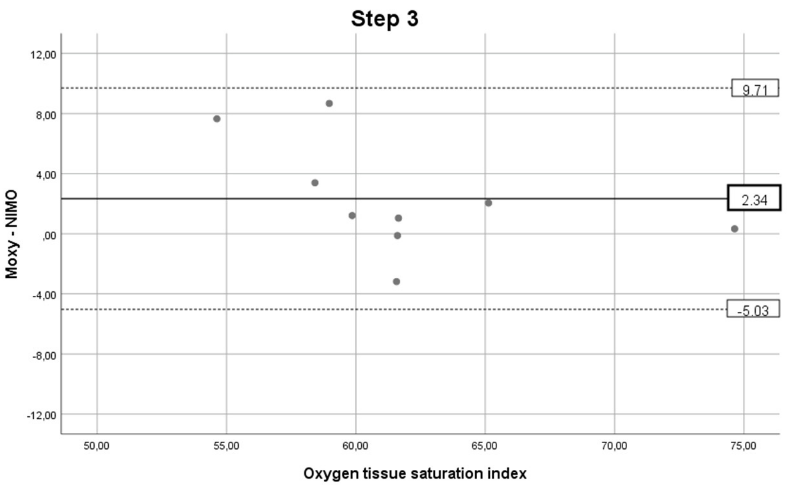
Step 3
Moxy - NIMO
Oxygen tissue saturation index
9.71
2.34
-5.03
12,00
8,00
4,00
,00
-4,00
-8,00
-12,00
50,00
55,00
60,00
65,00
70,00
75,00

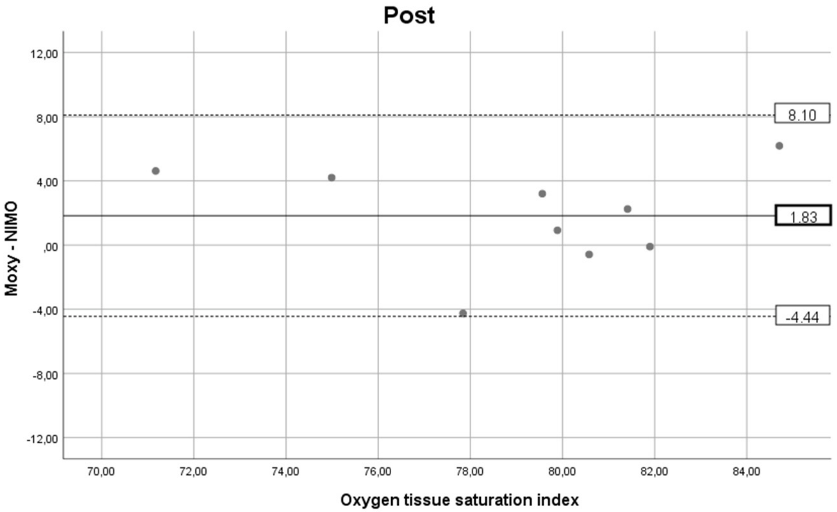
Post
Moxy - NIMO
Oxygen tissue saturation index
8.10
1.83
-4.44
12,00
8,00
4,00
,00
-4,00
-8,00
-12,00
70,00
72,00
74,00
76,00
78,00
80,00
82,00
84,00

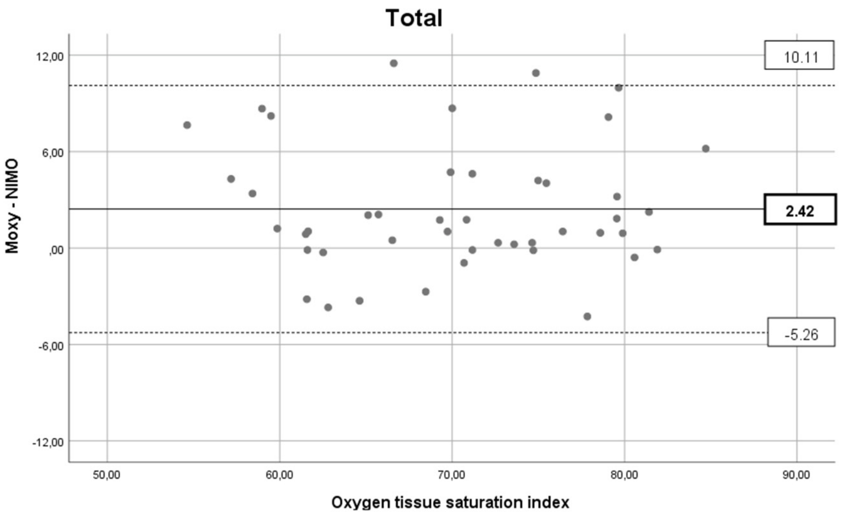
Total
Moxy - NIMO
Oxygen tissue saturation index
10.11
2.42
-5.26
12,00
6,00
,00
-6,00
-12,00
50,00
60,00
70,00
80,00
90,00
